# The surgical stabilization of multiple rib fractures using titanium elastic nail in blunt chest trauma with acute respiratory failure

**DOI:** 10.1007/s00464-015-4207-9

**Published:** 2015-04-15

**Authors:** Yih-Wen Tarng, Yuan-Yuarn Liu, Fong-Dee Huang, Hsing-Lin Lin, Tzu-Chin Wu, Yi-Pin Chou

**Affiliations:** Department of Orthopaedics, Kaohsiung Veterans General Hospital, Kaohsiung City, Taiwan; Division of Trauma, Department of Emergency, Kaohsiung Veterans General Hospital, 386, Da-Chung 1st Road, Kaohsiung City, 813 Taiwan; Research Center for Industry of Human Ecology, Chang Gung University of Science and Technology, Kweishan, Taoyuan, Taiwan; Department of Emergency, Fooying University Hospital, Pingtung County, Taiwan; Department of Medical Technology, Fooyin University, Kaohsiung City, Taiwan; Department of Nursing, Tajen University, Yanpu Township, Pingtung County, Taiwan; Division of Chest Medicine, Department of Internal Medicine, Chung Shan Medical University Hospital, Taichung, Taiwan; School of Medicine, Chung Shan Medical University, Taichung, Taiwan

**Keywords:** Rib fracture, Acute respiratory failure, Blunt chest trauma, Titanium nail, Surgical fixation

## Abstract

**Background:**

Blunt chest injuries are usually combined with multiple rib fractures and severe lung contusions. This can occasionally induce acute respiratory failure and prolong ventilations. In order to reduce the periods of ventilator dependency, we propose a less invasive method of fixing multiple rib fractures.

**Methods:**

Since October 2009, we have developed a new method to fix fractured ribs caused by blunt trauma. Rib fixations were performed using 2.0- or 2.5-mm intramedullary titanium elastic nails (TEN), with the help of video-assisted thoracoscopic surgery (VATS) and minimal thoracic incisions. All the patients’ demographics and postoperative data were collected.

**Results:**

From January 2010 to December 2012, a total of 65 patients presenting with multiple rib fractures resulting in acute respiratory failure were included in the study. Twelve patients received the new surgical fixation. Rib fixations were performed at an average of 4 days after trauma. Patients were successfully weaned off ventilators after an average of 3 days. The average length of stay in the hospital and the intensive care unit (ICU) was shorter for the patients with fixation than for nonsurgical patients. All twelve patients returned to normal daily activities and work.

**Conclusions:**

In the reconstruction of an injured chest wall, the VATS with TENs fixation in multiple rib fractures is feasible. This method is also effective in decreasing the length of the surgical wound. Because the structure of the chest cage is protected, the period of mechanical ventilation is shortened and the length of stay in the hospital and the ICU can be reduced.

Multiple rib fractures are usually found in patients with blunt chest trauma. These chest wall injuries can limit respiratory movements, especially in the presence of a flail chest. In addition to chest wall injuries, contusions of lung parenchyma are also frequently encountered. If the chest wall is destroyed, the probability of acute respiratory failure is increased [1, 2].

Ventilator support is still the first step in treating these patients. Although positive ventilation can establish internal pneumatic stabilization for the fractured segment of the ribs, this method can also cause pulmonary complications, including ventilator-associated pneumonia, empyema, or barotrauma [1, 3]. Shortening the period of ventilator dependency as much as possible is the most important treatment goal. Recently, operative stabilization of rib fractures has proved effective in the treatment of chest wall deformation caused by blunt trauma. Many fixation methods and materials have been studied, including wiring, a U-plate, a conventional long plate, intramedullary pin and Judet struts, etc [1, 4–6]. The advantages of these methods are that they provide a stable chest structure that maintains the expansion of the lung parenchyma. Stable chest walls allow continuous adequate ventilation and provide adequate tissue oxygenation, which reduces the need for mechanical ventilation [7–9]. Despite the many advantages of rib fixation, no definite fixation technique is widely accepted by most physicians, because all of these surgical methods require large thoracotomy wounds. Such surgical wounds can cause further destruction of the chest wall muscle, which can prolong the recovery time. All of these complications mitigate the benefits of the stabilization of fractured ribs.

Since 1990, video-assisted thoracoscopic surgery (VATS) has become a popular diagnostic and therapeutic tool for the treatment of hemodynamically stable patients with chest trauma [10–14]. In our hospital, VATS was usually used to manage retained pleural collections; however, fractured ribs were often ignored. Therefore, a new method was proposed for the stabilization of fractured ribs, using a titanium elastic nail (TEN) to fix the rib. This nail is an elastic intramedullary nail that is easily contoured, inserted, and removed. It allows adequate fixation for non-weight-bearing bones with a narrow medullary canal using three-point pressure on the canal, such as the clavicle or ribs [15]. Using VATS, definite incision wounds can be located. This study determines the benefits of fixing multiple rib fractures using TENs with VATS guidance and the success rate of the operation in patients with multiple rib fractures and respiratory failure.

## Patients and methods

### Patient selection

This study was conducted in a level-1 trauma medical center located in southern Taiwan. The center has approximately 15,000 emergency trauma visits per year. Patients with blunt thoracic injuries were included in this study. All of them were admitted to the emergency department (ED) and were managed under the guidelines of Advance Trauma Life Support (ATLS)^®^. Patients with multiple rib fractures (more than 4 ribs) accompanied by acute respiratory failure were enrolled. If hemothorax and pneumothorax were noted, tube thoracostomies were performed during the primary survey. All of these patients were initially hemodynamically stable, with no hypovolemic shock. They received complete secondary surveys in the trauma bay, including chest computed tomography (CT). Due to desaturation upon arrival at the hospital, emergency insertions of endotracheal tubes and ventilator support were performed at the trauma bay. Associated injuries were also evaluated. After initial management, patients were admitted to the ICU for further care.

All of these patients received ventilator support after admission. In order to wean them from the ventilator as soon as possible, surgical stabilization of the fractured ribs was designed. The inclusion criteria were patients between 18 and 75 years old and at least five rib fractures. The fractured sites of the ribs were located at the lateral and anterior regions of the chest. Because the main goal of this study was to determine the success of this new method of rib fixation, those patients with associated injuries that were too severe (Abbreviated Injury Score, AIS > 3) were excluded. Patients with severe medical conditions, such as liver cirrhosis, chronic heart or lung disease, chronic renal impairment, or a history of cancer, were all excluded. Patients who were hemodynamically unstable were also excluded.

### Surgical technique for intramedullary fixation using TENs

**Fig. 1:**
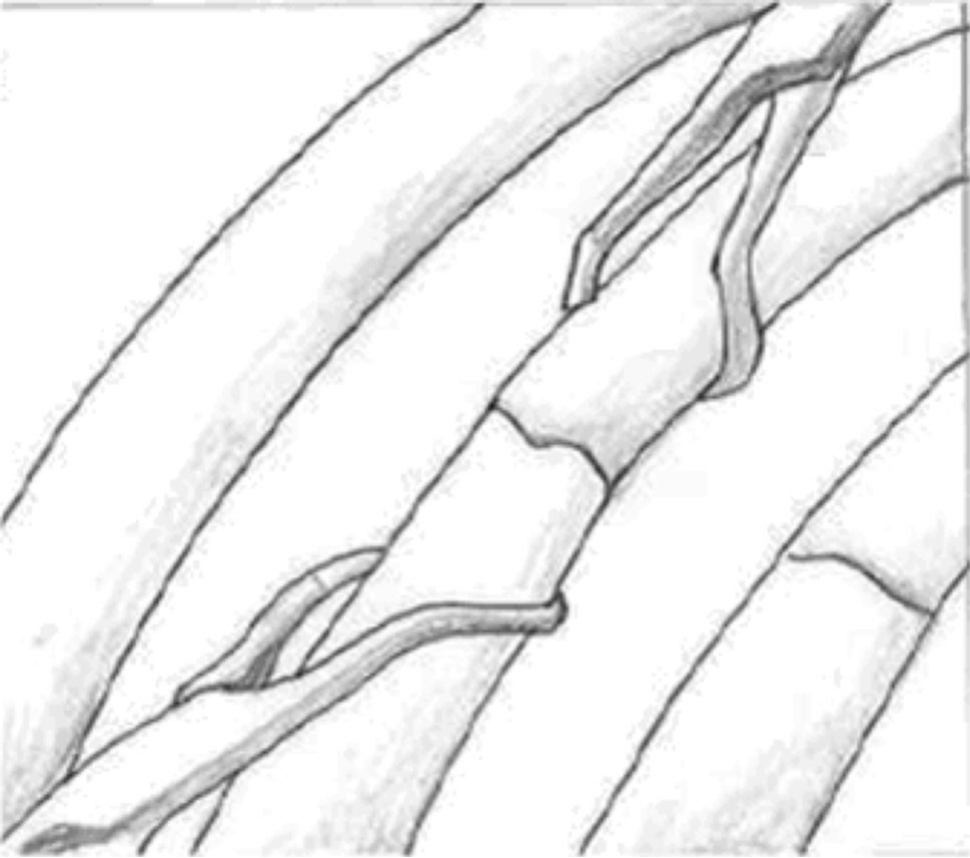
Redact the fractured rib closely using tower clips

**Fig. 2:**
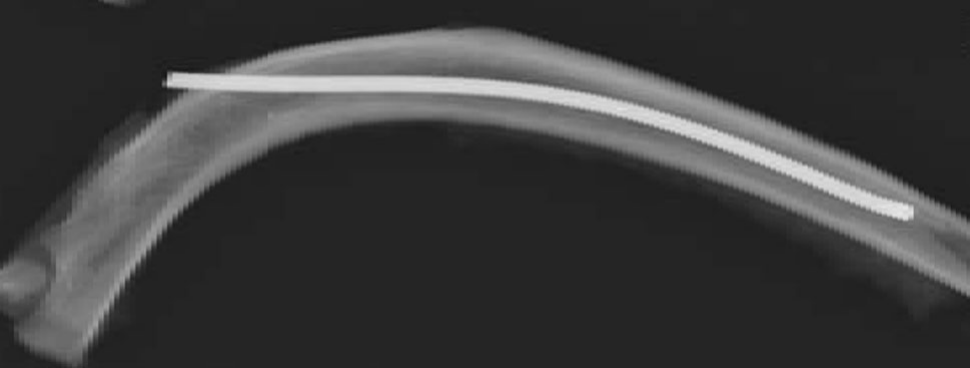
Titanium elastic nail (TEN) inserted in rib, providing three-point pressure on the inner cortex

**Fig. 3:**
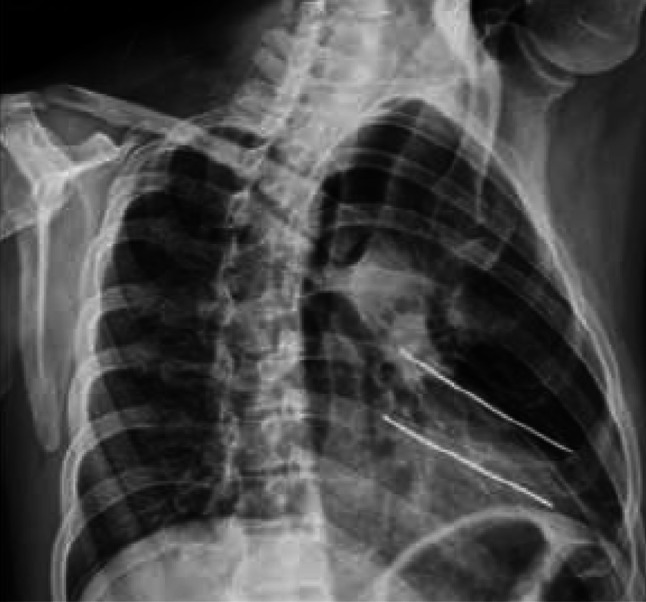
Patient receive TENs for fixation of fractured ribs

Patients received general anesthesia with double lumen endotracheal tube insertion. Each patient was placed in a true lateral position, with slight elevation of the operative site. The ipsilateral arms were abducted. The chest tubes previously inserted at the fifth intercostal spaces were removed. A 10 mm 0° thoracoscope was introduced via this old chest tube wound. Holding ventilation to force unilateral temporary lung collapse allowed a better view. The suction tube was introduced alongside the scope, to evacuate the pleural collection. The thoracoscope was then used to determine the fractured sites that were beneath the chest wall. A mini-transverse incision was made in the skin surface, above these fracture sites. The length was about 7–10 cm depending on the number of ribs requiring fixation. The serratus anterior was retracted anteriorly, and the latissimus dorsi was divided to expose the fracture site. The intercostal muscles were not divided. The fractured ribs were not retracted, and the parietal pleura remained intact. The TEN was used as the fixation materials. The fractured ends were redacted to a normal position by using tower clips (Fig. 1). Approximately 3–4 cm in front of the fracture site, an entry hole was created on the outer cortex of the fractured ribs, using a 2.0-mm drill and this hole was enlarged using an awl. The 2.0- or 2.5-mm TENs were precontoured by hand into the shape of a bow, with the tip of the nail pointing to the concave side of the bow. The TEN was then fixed in a universal chuck with a T-handle. Using oscillating hand movements, the surgeon gently advanced the unreamed TEN manually, until it reached the fracture site. The nail was introduced into the posterior fragment by direct manipulation of the fragment. The nail was then advanced along the intramedullary canal of the rib (Fig. 2). During this procedure, the thoracoscope was used to ensure that the nail did not penetrate into the pleural cavity and cause other injuries. The nail was advanced until it rested against the inner cortex of the opposite end. The nail was advanced manually throughout the entire implantation procedure to avoid penetration into the pleural cavity or the opposite outer cortex. The entry end of the nail was cut off at an appropriate length, at the site of its insertion. The cut end of the nail was embedded subcutaneously, beneath the muscle. A new chest tube was inserted. After surgery, all patients were admitted to ICU for further care. Chest roentgenograms were performed regularly to check the positions of the nails (Fig. 3).

### Postoperative care

All patients were subject to weaning-extubation programs for endotracheal tubes and ventilators, depending on the recovery of the lung. During this time, the chest tubes were connected to low-pressure suction (−15-cm H_2_O) to reduce the residual pleural space and to prevent early formation of blood clots. Chest tubes were removed when it was confirmed that there was no air leak, that there was fluid drainage less than 100 ml per day, and that the lung had completely expanded. Postoperative pain control began with a continuous intravenous (IV) morphine infusion, with a gradual shift to oral analgesics and muscle relaxants. Intramuscular injections of morphine were administered only at the patients’ request. After removal of the chest tube, the patients were discharged with tolerable pain, which was treated using oral analgesics at outpatient clinics (Table 1).

**Table 1:** Demographic analysis and clinical outcomes in patients (n = 12)

Mean age (year) (SD)	47.25 (14.37)
Males (%)	11 (91.6 %)
Mechanisms of injury	
Motorcyclist	11
Fall accident	1
Numbers of fractured ribs (mean, SD)	7.33 (1.15)
Pulmonary contusion score (mean, SD)	6.25 (1.05)
Anatomic injury score (AIS) of thoracic injury (mean, SD)	3.75 (0.45)
Associated injuries	
Head injury (%)	6 (50.0 %)
Abdominal injury (%)	4 (33.3 %)
Extremity injury (%)	10 (83.3 %)
Multiple trauma	11 (91.7 %)
ISS (mean, SD)	21.17 (4.13)
Time from trauma to perform VATS (days) (mean, SD)	3.83 (0.83)
Numbers of ribs fixed by TENs (mean, SD)	2.58 (0.51)
Time of ventilator use (days) (mean, SD)	6.42 (0.79)
Time of ventilator use after rib fixations (days) (mean, SD)	2.5 (0.67)
Time of chest tube use (days) (mean, SD)	10.50 (1.17)
ICU length of stay (days) (mean, SD)	8.00 (0.95)
In-hospital length of stay (days) (mean, SD)	15.17 (2.69)

### Data collections and postoperative follow-up

The time spent on the ventilator, the duration of chest tube use, and the length of stays in the ICU and in the hospital were all recorded. When fever occurred, microbial cultures were performed by collecting the sputum and discharge from the chest tube. The patient’s age and gender as well as the mechanism of the injury were all recorded.

After discharge from the hospital, routine follow-up was scheduled for the first week, the second week, the sixth week, the third month, the sixth month, and then annually. The mean follow-up period was 21 months (range 18–24 months). The fracture union was determined using plain radiographs, which were taken immediately after surgery and at 6-week intervals, for 6 months. The location of the TENs was checked again using rib-detail radiographs that were taken every year, postoperatively. All of these procedures were approved by the Institutional Review Board of the Kaohsiung Veterans General Hospital.

## Results

From January 2010 to December 2012, a total of 728 patients were admitted to the ED because of blunt chest trauma. Sixty-five patients had multiple rib fractures with acute respiratory failure. The penetrations of the fractured ends into the pleural cavity and resulting chest wall deformation were noted in these patients. Fifteen patients were initially enrolled in the study. Three of these were excluded because they exhibited massive hemothorax with unstable vital signs, which necessitated an emergency exploratory thoracotomy. Almost all of the patients were the victims of motorcycle accidents, except for one fall injury. Only one patient was female. The age range was 22–67 years old (the mean age was 47.25, SD = 14.37). All patients underwent a chest tube thoracostomy in the ED because of traumatic hemothorax and pneumothorax. Pulmonary contusion scores were calculated using chest X-ray initially, upon arrival at the hospital, and 24 h after trauma. The average contusion score was 6.25 (SD = 1.05). The average AIS for the chest was 3.75 (SD = 0.45). The average injury severity score (ISS) was 21.17 (SD = 4.43). Table 1 shows all of the demographic characteristics of the patients.

All patients were intubated with endotracheal tubes within 4 h after trauma because of acute respiratory failure. Only one patient exhibited pure chest trauma. Most patients presented with multiple injury sites. Associated injuries included six head injuries, four abdominal injuries, and ten extremity injuries. The average time period between the trauma and the operations was 3.83 days (SD = 0.83).

The number of rib fractures was calculated using a chest CT. The mean number of fractured ribs was 7.33 (SD = 1.15). Nine patients had flail chest. Not all of the fractured ribs required fixation. The mean number of ribs fixated using TENs was 2.58 (SD = 0.51). Clavicle fractures were noted in 10 patients who had upper-rib fractures. In order to provide a more stable structure for the upper chest, the clavicle fractures were all fixed simultaneously, during surgery for the ribs. Five patients had a scapular fracture. These fractures resulted from back contusion, and all were managed with conservative treatment.

All of the patients were weaned off the ventilator and extubated smoothly within 3 days after surgery (average 2.5 days, SD = 0.67), depending on the recovery from lung contusion. The average period for ventilator use was 6.42 days (SD = 0.79). No patients required a tracheostomy. The mean length of stay in the ICU was 8.0 days (SD = 0.95). The average length of stay in the hospital was 15.17 days (SD = 2.69).

During this study period, the data of non-fixation for fifty-three patients were also collected. The mean age of the non-fixation group was 58.28 (SD = 19.01), which was much higher than the mean age of the fixation group (*p* *=* 0.035). Thirty-one patients in this group had brain injury with hemorrhage. All of them received conservative treatments without craniotomy. Twenty-eight patients also had intra-abdominal bleeding, and half of them required surgical intervention. Although the mean AIS of chest (average 3.49, SD = 0.58) and pulmonary contusion scores (average 5.92, SD = 1.21) were similar in both groups, the higher percentage of associated injuries resulted in a higher ISS in the non-fixation group (average 26.09, SD = 5.96). Nineteen patients in this group received VATS evacuation because of retained pleural collections. Due to multiple complicated associated injuries in this group, the period of ventilator dependence was prolonged (average 19.30 days, SD = 16.35). The length of stay in hospital (average 35.55 days, SD = 19.46) and in ICU (average 16.70 days, SD = 9.62) was also much longer than for the rib fixation group.

There was no surgical mortality in this study. Long-term regular follow-up was performed for all patients. The rib fractures united uneventfully within 3 months, without any migration or backing out of the TENs in the first year. No patients required nail removal or complained of evident pain at the operative site for TEN placements.

## Discussion

Rib fractures are usually incurred in blunt chest trauma. Severe chest pain that results from fractured ribs prevents patients from breathing deeply [1, 16]. As a result of hemothorax or pneumothorax, the lung parenchyma collapses. In addition to chest wall deformation, lung contusions are also frequent. The alveolar sac fills with blood because the capillaries of the alveoli hemorrhage. These complications can induce acute respiratory failure. If the period of dependency on a ventilator is prolonged, the risk of infection increases. These post-trauma infections lead to an increase in the length of stay in the ICU and in the hospital. The rates of morbidity and mortality also increase.

Pain control is the first step in treating multiple rib fractures. Active pain relief improves respiratory movement and prevents hypoxia [2, 17]. Oral or intravenous analgesics, including narcotics and nonsteroidal anti-inflammatory drugs, are usually used. These agents are usually ineffective in patients with multiple rib fractures. More invasive techniques, such as epidural analgesia or nerve block, are often required in these cases. Sometimes, these methods also fail, because movement of fractured ribs causes persistent pain and a gradual decrease in respiratory movements. Multiple rib fractures deform the chest cage, so total lung volumes are decreased. To address this problem, several materials, such as rib tape or belts, are applied to the surface of the chest to fix fractured ribs [18]. These methods are only partially effective in the fixation of fractured ribs and are not commonly used by most physicians.

The surgical fixation of fractured ribs has been studied since 1980. Several studies have reported that surgical fixation is feasible and effective in stabilizing fractured ribs [7–9, 19–21]. Of these studies of rib fixation, the strongest evidence of the benefit of reducing the ventilation period was provided by Tanaka et al. [22]. This prospective study showed that rib fixation can result in a significant reduction in the period of ventilator dependency. Although these results are encouraging, routine rib fixation is still a controversial practice. There are several reasons for this. First, the ribs are surrounded by intercostal muscle groups. The fractured ends of the ribs usually contact each other and heal spontaneously. Second, a rib is not a weight-bearing bone. Its main function is to maintain the chest’s structure and shape. If there are multiple rib fractures with acute respiratory failure, positive ventilation can expand the lung sufficiently, even if the chest wall is destroyed. Third, no procedure-specific orthopedic materials are available for fractured ribs. Two surgical materials are commonly used for rib fixation: a traditional screw with a plate and Kirschner’s wire. The use of screws and plates for fractured ribs requires an extended thoracotomy [4, 22, 23]. Extensive incisions can cause more pain and further destruction of the muscle layers. During the fixation period, only one layer of the cortex of the ribs is engaged in order to avoid irritation of the parietal pleura under the ribs. This can result in insecure fixation of the fractured ribs. Kirschner’s wire is used as an intramedullary nail to fix fractured ribs [5–7]. This wire is a straight, smooth, rigid stainless steel pin that has no resistance to a bending force. Although this method requires a smaller incision wound than a plate and screw, the exact fracture sites must be determined during an exploratory operation. This wire has a sharp tip that can easily penetrate the outer cortex of a rib. A Kirschner’s wire also does not provide three-point pressure on the inner surface of the rib’s bone marrow. This can lead to migration or backing out from the fixed ribs. These two disadvantages of Kirschner’s wire mean that there is a high risk of injury to the surrounding soft tissues or vital organs.

In the study hospital, a different method is used to fix the fractured ribs. In order to decrease the length of the surgical wound, video-assisted thoracoscopy (VATS) is used to determine the fracture sites from the pleural cavity. The clear view provided by VATS allows a smaller incision wound, just above the fracture site, which reduces the area of muscle destruction. VATS is also used to evacuate the retained pleural collections. VATS is also important in monitoring the procedure for fixation. Ribs are non-weight-bearing bones. Their main function is to maintain the shape of the chest wall in order to allow the lungs to expand. Anatomic reduction of a fractured rib is not necessary. Because the use of VATS allows for smaller wounds, the intra-medullary nail is the best choice. Rather than Kirschner’s wire, TENs are used for fixation. This system was originally designed for diaphyseal fractures of the long bones in children [24]. In a previous study, one pre-bent TEN was successfully inserted into the clavicle by the medullar canal to provide three-point pressure on the inner cortex of the bone [15]. The anatomic characteristics of the ribs and the clavicle are similar. Both have a narrow canal and are curved. Experience in the fixation of the clavicle with TEN systems demonstrates that an elastic nail can exert the same three-point pressure on a rib. Unlike Kirschner’s wire, a TEN has a hockey-stick-like tip, which does not penetrate the outer cortex of the rib. This stick also prevents migration or backing out from the fixed ribs and into the surrounding soft tissues. The elastic characteristics of a TEN give three-point pressure on the inner surface of the bone. This also prevents migration from the bone marrow canal.

During the study period, all of the operations were uneventful. There were no surgical complications and no surgical mortalities. The length of the surgical wound was limited to 8–12 cm. The first three patients had larger surgical wounds because the surgeon was unpracticed in the technique. However, even during this learning period, no patients required a standard exploratory thoracotomy. In this early period, the most important details of this operation were identified. First, the fracture sites of the ribs must be evaluated carefully. Based on the anatomic distribution of the twelve pairs of ribs, they are divided into three groups: the first to the third ribs, the fourth to the ninth ribs, and the tenth to the twelfth ribs. Rib fractures in the first to the third ribs do not cause a deformation of the chest cavity, because the thick muscle layers of the shoulder girdle are located on the lateral and posterior sides, and the pectoris major and minor are located on the anterior side. All of these muscle groups prevent severe deformation of the chest during accidents. The clavicle bone, located at the apex of the chest wall, also provides excellent support for the upper chest. If the clavicle bone is fractured and there is total displacement, fixation of the fractured clavicle bone to support the upper chest is recommended. The thick muscle layers around the first to the third ribs also make any approach to the upper chest difficult. A larger incision wound is required, which means that the large chest wall muscle groups are destroyed. The tenth to the twelfth ribs are located beneath the diaphragm. Fractures in this region do not degrade the integrity of the chest cavity, and there is no need for fixation. The region of the fourth to the ninth ribs comprises the largest volume of the entire chest cavity. Fractures in this region can cause severe deformation of the chest wall, especially when combined with flail chest. Rib fractures in this region are suitable for fixation. Second, only fractures on the anterior lateral side can be fixed, because injuries in this region usually cause severe chest wall deformation. The posterior third ribs, especially near the thoracic vertebral spine, do not require fixation. Ribs in these areas are surrounded by para-spinal muscle groups. These muscle groups are thick and strong. Fractures in these regions are protected by these muscle groups, and severe deformation of the chest wall is prevented. This strong musculature not only inhibits movement of the fracture site, but also provides an adequate blood supply for rapid healing. Any approach to these regions is also very difficult, because of these strong para-spinal muscles. Third, not all fractured ribs require fixation. The tenting force of a fixed rib can cause the unfixed rib to return to its original position spontaneously. An example of this is when a patient has continuous fractures to the fourth to the eighth ribs. Not all five ribs require fixation. It is possible to fix the fourth, sixth, and eighth ribs only. The other two fractured ribs are then pushed to their primary position, which maintains the stability of the chest wall. This procedure reduces use of materials and the total time required for surgery.

Although there are several advantages to the fixation of fractured ribs using TENs, this study has some limitations. Since the main goal of this study was to determine the safety of TENs in fractured ribs when there is acute respiratory failure, the candidates for this procedure were chosen very carefully. The total number of patients is too small, which is a problem because less than ten percent of blunt chest trauma patients experience acute respiratory failure, so only a few patients could be enrolled in this study. In fact, 65 patients with multiple rib fractures presented with acute respiratory failure during this three-year period, but only 12 patients were suitable candidates for this procedure. There are several factors to influence our trauma surgeon making this surgical decision. Age is the first important factor to be considered. Patients older than age 70 are not suitable candidates for rib fixation using TEN systems. In older patients, the cortex of the ribs is thinner. Even though these intramural nails are elastic, the possibility of perforating the side of the ribs is increased. In our study, the mean age of non-fixation patients was higher than that of the fixation group (58.28 vs. 47.25, *p* *=* 0.035). Associated injuries are another important factor. Even though the AIS of the chest in both groups was the same (3.75 vs. 3.49, *p* *=* 0.11), the non-fixation group had a higher percentage of head or abdominal injuries. The injury severity score in the non-fixation group was slightly higher than in the fixation group (26.09 vs. 22.33, *p* *=* 0.051). Fractured sites of ribs could also influence the decision regarding fixation. The fixation group had a higher percentage of lateral side fractures (100 vs. 43 %). All these factors show obvious differences between these two groups. The clinical outcomes in the fixation group were better than in others. The mean periods of ventilator dependency were shorter for the surgical group (6.42 vs. 11.35 days). The length of stay in ICU and in hospital was also shorter for the surgical group (7.33 vs. 16.7 days in ICU; 15.17 vs. 35.55 days in hospital). Although these results have no statistical meaning because there is too much bias between the two groups, there is tentative evidence that the fixation of fractured ribs results in better clinical outcomes. Patients with unstable vital signs are also not suitable candidates for VATS procedures. These patients must be managed with a standard emergency thoracotomy to check for bleeders. Therefore, the number of cases in this study is decreasing. Suitable surgical material is another limitation. The TEN-fixation system was initially used for long-bone fractures in children. It is not specifically designed for rib fractures. An important problem is backing out of the nail because the canal is smaller in the ribs. Even the thinnest nail can be pushed out because of the higher pressure in the narrowing bone marrow cavity of the ribs. Three patients in this study experienced this minor post-traumatic complication. All three patients had an associated head injury. During the postoperative period of ventilator dependency, patients became irritable when sedative drugs were withheld, and the nails were pushed out by violent movement. To prevent this complication, these patients should have received ventilation under sedation for a longer time. The search for other suitable materials is ongoing. There are also other problems. The TEN is preserved in the ribs and is not removed, even when the fractured bone has healed. All of the patients were tracked for at least 24 months. A chest roentgenogram was obtained each month. This frequent follow-up results in increased expense for the patient and the National Health Insurance system.

## Conclusion

Fixation by TEN in patients with multiple fractured ribs is a safe and effective method of maintaining the structure of the chest cavity. A larger and more prospective protocol is required to confirm the real benefits to the clinical outcome.
